# Individual and Conjoint Factors Associated With Beliefs About the Harmfulness of Nicotine Replacement Therapies Relative to Combustible Cigarettes Among People Who Smoke: Findings From the 2020 ITC Four Country Smoking and Vaping Survey

**DOI:** 10.1093/ntr/ntad075

**Published:** 2023-05-17

**Authors:** Hua-Hie Yong, Chandan Karmakar, Mohammod Abdul Motin, Ron Borland, K Michael Cummings, Shannon Gravely, Geoffrey T Fong

**Affiliations:** Deakin University, Geelong, VIC, Australia; Deakin University, Geelong, VIC, Australia; Rajshahi University of Engineering and Technology, Bangladesh; University of Melbourne, VIC, Australia; Medical University of South Carolina, Charleston, SC, USA; University of Waterloo, Waterloo, ON, Canada; University of Waterloo, Waterloo, ON, Canada; Ontario Institute for Cancer Research, Toronto, ON, Canada

## Abstract

**Introduction:**

This study examined individual and conjoint factors associated with beliefs about the harmfulness of nicotine replacement therapies (NRTs) relative to combustible cigarettes (CCs).

**Aims and Methods:**

Data analyzed came from 8642 adults (≥18 years) who smoked daily/weekly and participated in the 2020 ITC Four Country Smoking and Vaping Survey in Australia (*n* = 1213), Canada (*n* = 2633), England (n = 3057), and United States (*n* = 1739). Respondents were asked: “Compared to smoking cigarettes, how harmful do you think nicotine replacement products are?” Responses were dichotomized into “much less” versus otherwise for analysis using multivariable logistic regression models, complemented by decision-tree analysis to identify conjoint factors.

**Results:**

Percentages believing that NRTs are much less harmful than CCs were 29.7% (95% CI = 26.2% to 33.5%) in Australia, 27.4% (95% CI = 25.1% to 29.8%) in England, 26.4% (95% CI = 24.4% to 28.4%) in Canada, and 21.7% (95% CI = 19.2% to 24.3%) in the United States. Across all countries, believing nicotine is not at all/slightly harmful to health (aOR = 1.53–2.27), endorsing nicotine vaping products (NVPs) as less harmful than CCs (much less harmful: aOR = 7.24–14.27; somewhat less harmful: aOR = 1.97–3.23), and possessing higher knowledge of smoking harms (aOR = 1.23–1.88) were individual factors associated with increased odds of believing NRTs are much less harmful than CCs. With some country variations, these nicotine-related measures also interacted with each other and sociodemographic variables to serve as conjoint factors associated with the likelihood of accurate NRT relative harm belief.

**Conclusions:**

Many people who regularly smoke cigarettes are unaware that NRTs are much less harmful than cigarettes. Additionally, beliefs about NRTs relative harmfulness appear to be influenced by both individual and conjoint factors.

**Implications:**

This study demonstrates that despite past efforts to educate people who smoke about the harms of NRTs relative to CCs, misperceptions around the relative harmfulness of NRTs remain substantial. In all four studied countries, subgroups of people who smoke regularly who are misinformed about the relative harmfulness of NRTs, and who may be reluctant to use NRTs for smoking cessation can be reliably identified for corrective interventions based on their understanding of the harms related to nicotine, NVPs and smoking along with sociodemographic markers. The identified subgroup information can be used to prioritize and inform the development of effective interventions to specifically address the gaps in knowledge and understanding of the various subgroups identified. Our results suggest these may need to be tailored for each country.

## Introduction

The risk profiles of different nicotine-containing products are not all the same.^1^ Compared to combustible cigarettes (CCs), nicotine replacement therapy products (NRTs) are substantially less harmful^1,2^ and recent evidence indicates that NRTs are still one of the most commonly used smoking cessation aids.^3^ However, a non-trivial number of people who currently smoke misperceive NRT as equally or more harmful than CCs.^4–6^ One possible reason for the misperception of the relative harmfulness of NRT relates to the mistaken belief that nicotine is the most harmful constituent of tobacco.^4–7^ This is consistent with the finding that belief about nicotine harmfulness is a strong predictor of perceptions of NRT product harmfulness relative to CCs.^8^ Recent research from our team also indicates that harm perceptions of NRT and nicotine vaping products (NVPs) relative to CCs are highly correlated.^9^ This correlation implies that different types of nicotine-containing products may be perceived similarly in terms of their risk profiles relative to CCs, of which tobacco smoke contains over 7000 chemicals, with at least 250 are known to be harmful and 69 can cause cancer (eg, tobacco-specific nitrosamines, formaldehyde).^10^ On the spectrum of risk related to nicotine products, NRT is considered the least harmful, with cigarettes being the most harmful.^1^ However, the lack of discrimination in relative harm perception of different types of nicotine-containing products among people who smoke, and whether or how misperception of the harm of one nicotine product influences another, warrants further investigation.

One consequence of misperceptions of the relative harmfulness of NRT is that they are undermining the use of these efficacious, government-approved products for smoking cessation,^11–13^ and among those who have used them for quitting purposes, they may not be using them properly to benefit from the therapeutic dose and duration recommended.^5,12,14^ Thus, it is important that these misperceptions are addressed to ensure that people who smoke can make informed choices about whether and when NRT should be used to help them stop smoking, and if choosing to use NRTs, that they can use them properly.

Past research has indicated that perceptions around NRT harms relative to smoking are influenced by factors such as age, gender, ethnicity, educational attainment, and past use of any nicotine medications.^4,5^ However, whether and how these factors might interact to influence relative harm perception of NRTs is somewhat unclear. Previous investigations have examined this relationship using regression-based approaches, which typically do not test for interactions between predictor variables on outcomes beyond a two-way interaction because of the difficulty in interpreting higher-order interactions (eg,^15^). An alternate analytic strategy such as decision-tree analysis (DTA) can be used to complement the commonly used conventional regression approach as it can efficiently handle higher-order complex interactions. DTA does this by creating binary segmentations of individuals into subgroups based on their responses to a set of predictor variables resulting in a tree-like structure called decision tree which is intuitive and highly interpretable.^16,17^ This novel approach has the potential to shed light on what factors conjointly influence people’s perceptions and beliefs about the harms of different nicotine-containing products, which then allows interventions to be tailored for subgroups rather than applying them generically across the populations.

The regulatory policies for accessing NRT vary across the four countries studied herein, even though they all share similar guidelines recommending the use of combination NRT for smoking cessation.^18–20^ Obtaining NRT via prescription is rare in Australia, Canada, and the United States, but appears to be more common in England largely because in England, NRT prescriptions can be issued by a general practitioner or other prescribers for a small prescription fee, or free of charge for some groups of patients through the National Health Service.^21–25^ However, in England, NRT is more commonly purchased over the counter from pharmacies, supermarkets, and other outlets.^24^ In Australia, NRT products can be purchased over the counter from pharmacies (at a subsidized rate if prescribed by a doctor) and some from supermarkets.^26^ In Canada, NRT is available over the counter; but there is no national reimbursement scheme for NRT use. However, some Canadian provinces subsidize NRT, and workplace insurance programs may cover prescriptions.^27^ In the United States, the NRT gum, patch, and lozenge are available as either over the counter or on prescription, but nasal spray and inhaler are only available on prescription.^28^ The differing policies on NRT accessibility and subsidization across countries may affect perceptions of the harm of NRT and in turn, the extent of NRT use by people who smoke.

Making use of two complementary analytic strategies, namely traditional logistic regression and DTA, and data available from England, Australia, Canada, and the United States, among people who smoke daily or weekly, this study aimed to: (1) identify key factors individually and/or conjointly associated with the harmfulness beliefs about NRT use relative to CCs, and (2) determine whether there were any country differences.

## Methods

### Sample and Design

Data for this cross-sectional study were from the 2020 ITC Four Country Smoking and Vaping (ITC 4CV) Survey, conducted in Australia, Canada, England, and the United States. The sample was limited to people who currently smoke cigarettes (at least weekly), regardless of any use of any other nicotine products, which resulted in 8642 respondents. Sample characteristics are presented in **Table 1**. Details of the study design and methodology have been reported elsewhere.^29^ Briefly, the ITC 4CV survey began in 2016 and participants consisted of persons aged ≥18 years who smoke either recontacted from the original ITC 4C cohort^30^ or newly recruited from online panels using either probability-based sampling frames, non-probability opt-in panels or a combination of these. Participants were recontacted to complete the online survey in 2018 and 2020. New participants were recruited to address attrition and maintain sample size over time.

**Table 1.** Sample Characteristics by Country

Variables	Canada *N* = 2633	United States *N* = 1739	England *N* = 3057	Australia *N* = 1213
Age group in years [% (*n*)]				
18–24	22.6 (594)	22.8 (396)	25.1 (766)	1.2 (14)
25–39	23.5 (618)	15.7 (273)	21.6 (659)	14.8 (180)
40–54	25.3 (665)	19.4 (337)	25.1 (768)	31.7 (384)
≥55	28.7 (756)	42.2 (733)	28.3 (864)	52.4 (645)
Female [% (*n*)]	52.5 (1382)	51.7 (899)	46.8 (1430)	47.8 (580)
Income [% (*n*)]				
Low	28.9 (761)	36.1 (627)	20.9 (639)	28.2 (342)
Medium	27.7 (729)	28.8 (501)	32.2 (984)	21.1 (256)
High	37.2 (980)	34.7 (604)	40.1 (1226)	44.3 (537)
No Information	6.2 (163)	0.4 (7)	6.8 (208)	6.4 (78)
Education [% (*n*)]				
Low	28.9 (757)	36.6 (636)	13.3 (399)	30.3 (366)
Medium	44.1 (1154)	41.3 (718)	54.0 (1620)	41.1 (497)
High	27.0 (707)	22.1 (384)	32.7 (982)	28.6 (346)
Ethnicity [% (*n*)]^				
Ethnic majority/English speaking	80.6 (2090)	70.2 (1219)	88.6 (344)	89.2 (1079)
Ethnic minority/non- English speaking	19.4 (502)	29.8 (518)	11.4 (2673)	10.8 (131)
Smoking status [% (*n*)]				
Daily smoking	81.8 (2153)	84.1 (1462)	83.4 (2549)	94.2 (1143)
Weekly smoking	18.2 (480)	15.9 (277)	16.6 (508)	5.8 (70)
Vaping status [% (*n*)]				
Daily vapers	14.1 (372)	14.0 (244)	20.4 (625)	5.9 (72)
Non-daily vapers	21.0 (552)	14.6 (253)	20.7 (633)	8.0 (97)
Non-vapers	64.9 (1709)	71.4 (1242)	58.9 (1799)	86.1 (1044)
Knowledge of smoking health effects Mean (SD), range = 0–4	1.26 (0.71)	1.02 (0.70)	1.24 (0.76)	1.45 (0.76)
Belief re harmfulness of nicotine to health [% (*n*)]				
Not at all harmful	2.8 (74)	3.0 (51)	4.7 (143)	4.1 (49)
Slightly harmful	18.9 (497)	17.4 (301)	23.1 (704)	18.9 (228)
Moderately harmful	31.5 (828)	35.0 (604)	32.8 (1001)	33.4 (404)
Very harmful	31.5 (827)	26.4 (456)	22.0 (671)	25.2 (304)
Extremely harmful	11.6 (306)	12.6 (217)	11.3 (344)	11.6 (140)
Don’t Know	3.7 (97)	5.7 (98)	6.2 (188)	6.9 (83)
Belief re harmfulness of vaping relative to smoking [% (*n*)]				
Much less harmful	8.3 (215)	7.8 (134)	13.7 (415)	11.2 (134)
Somewhat less harmful	29.9 (776)	23.6 (406)	38.0 (1148)	28.8 (344)
Equally harmful	38.1 (990)	38.5 (663)	28.3 (857)	27.2 (325)
Somewhat more harmful	8.2 (213)	9.7 (167)	6.4 (194)	4.5 (54)
Much more harmful	5.2 (134)	7.0 (120)	2.5 (76) 11.1 (335)	2.9 (35) 25.3 (302)
Don’t Know	10.5 (272)	13.5 (232)		

percentages and frequencies are unweighted; SD = standard deviation;

Income and education were coded into low, moderate, and high categories to equate them across countries. Household annual income was categorized into low (< AU$45 000 in Australia, < CA$45 000 in Canada, < US$30 000 in the United States, and < £15 000 in England), moderate (AU$45 000–74 999, CA$45 000-74 999, US$30 000–59 999, and £15 000–39 999) or high (≥ AU$75 000, ≥ CA$75 000, ≥ US$60 000, and ≥ £40 000), plus a No information category to include those who refused to answer this question. Low education was defined as having completed high school or less in Australia, Canada and the United States, or secondary, vocational or less in England; moderate education referred to some university (no degree), community college, technical school and trade in Canada and the United States, some university (no degree), technical school and trade in Australia, or college and university (no degree) in England; and participants in all countries who completed university or postgraduate were considered to have high education.

^, Respondents were classified as being in the ethnic majority group if they were white (United States, Canada, England) or spoke English in the home in Australia and were defined in the ethnic minority group otherwise;

Distribution of all variables show country differences significant at *p* < .001;

### Measures

#### Outcome

Harm perception of NRT products relative to CCs was assessed using the question: “Compared to smoking cigarettes, how harmful do you think nicotine replacement products are?” with a clarifying note that nicotine replacement products include patches, gum, inhalers, mouth spray, and various other nicotine products that have been approved as medicines. Response options included^1^: Much less..,^2^ Somewhat less..,^3^ Equally..,^4^ Somewhat more..,^5^ Much more.., harmful than smoking cigarettes. For analysis purposes and consistent with known risk profile of NRT products on the spectrum of risk of nicotine-containing products,^1^ responses were dichotomized into “much less harmful” (defined as the “correct” perception) versus “other” (“incorrect” perception which included somewhat less harmful, equally harmful, somewhat more harmful, much more harmful and also don’t know responses) as per past research.^15^

#### Predictors

Age groups (18–24, 25–39, 40–54, and ≥55), gender (male, female), education (low, moderate, or high), income (low, moderate, high, or No information), ethnicity (ethnic majority vs. minority), country, smoking frequency (daily versus weekly) and current vaping status (daily, non-daily, and not currently vaping), nicotine harmfulness belief (“How harmful do you think nicotine is?”; response options were not at all, slightly, moderately, very, or extremely harmful but as response distribution was highly skewed, responses were dichotomized into Not at all/Slightly harmful versus Moderately/Very/Extremely harmful/don’t know for analysis), belief about the harmfulness of NVPs relative to CCs (“Compared to smoking cigarettes, how harmful do you think vaping (using e-cigarettes) is?” with response options: Much less harmful, somewhat less harmful, equally harmful, somewhat more harmful, or much more harmful; for analysis purposes responses for the equally and more harmful categories were combined to represent the “incorrect” belief and also to increase cell size), and knowledge of health effects of smoking (derived by summing the Yes responses to the 4 parallel questions: “Based on what you know or believe, does smoking cause—stroke in smokers; blindness; breast cancer; mouth cancer?).

### Data Analysis

Two complementary analytic approaches were undertaken. The first involved multivariable logistic regression modeling conducted in a stepwise fashion using Stata Version 15 to: (1) examine the unadjusted and adjusted association of each predictor with the belief that NRTs are “much less harmful” than CCs, and (2) explore possible country differences by testing for predictor by country interactions. As significant by-country interactions were found, country-stratified analyses were undertaken to aid interpretation of country differences.

To compliment the first approach, the second approach involved a DTA to explore the possibility of more complex interaction and nonlinear effects in order to understand how these factors might interact with each other to conjointly influence NRT relative risk perception. DTA was conducted separately by country for ease of interpretation and to determine presence of any country differences. Supplementary Figure S1 presents the steps involved in DTA. The initial sample was randomly split into a training set (70%) and a test set (30%). The training dataset was used for developing decision-tree models using decision-tree classifiers to generate optimal decision-tree models to best represent the different subgroups identified. To reduce bias in the learning task, the learning set was first subject to a class balancing process^31^ before being used for training and validation using a 10-fold method where the model with the highest area under the curve value of receiver operating characteristics was selected for the final tree model. This model was then evaluated for its performance on the test set data from within and between countries. All classification analyses were conducted using MATLAB R2018b software.

## Results

**Figure 1.**
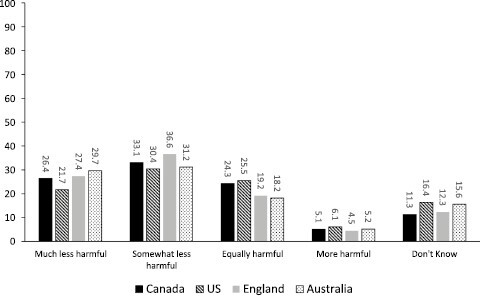
Weighted percentage estimates of reported belief regarding nicotine replacement therapy harmfulness relative to cigarette smoking among people who currently smoke daily/weekly by country.

Across the four countries, beliefs about NRTs being much less harmful than CCs were lowest in the United States (weighted estimate = 21.7%) and highest in Australia (29.7%), with Canada (26.4%) and England (27.4%) in between (see **Figure 1**). Overall, across the four countries, the percentage endorsing either equally harmful, more harmful, or don’t know ranged from 36% in England to 48% in the United States. Of note, among the different sets of beliefs about NRT, a majority in all four countries believed that NRTs are somewhat less harmful than CCs, with the United States again the lowest (30.4%) but England the highest in endorsing this belief (36.6%).

Factors independently associated with the belief that NRTs are much less harmful than CCs.

Multivariable logistic regression revealed some country differences in findings, thus, results are presented separately by country (see **Table 2**; see Supplementary Table S1 for unadjusted results).

**Table 2.** Multivariable Logistic Regression Results Showing Factors Associated With Belief About Nicotine Replacement Therapy Products Being Much Less Harmful Than Combustible Cigarettes Among People Who Currently Smoke Daily/Weekly

Variables	Canada *N* = 2549	United States *N* = 1706	England *N* = 2935	Australia *N* = 1183
	aOR (95% CI)	aOR (95% CI)	aOR (95% CI)	aOR (95% CI)
Gender				
Female	1.06 (0.88 to 1.29)	0.88 (0.69 to 1.13)	0.96 (0.80 to 1.15)	1.17 (0.88 to 1.54)
Male	Ref	Ref	Ref	Ref
Age group^	Ref	Ref	Ref	Ref
18–24	1.03 (0.77 to 1.38)	1.32 (0.87 to 2.02)	0.87 (0.66 to 1.15)	1.72 (0.39 to 7.55)
25–39	1.39 (1.04 to 1.86)*	1.47 (0.97 to 2.23)	0.92 (0.71 to 1.20)	2.44 (0.57 to 10.48)
40–54	1.65 (1.23 to	1.93 (1.32 to	0.82 (0.63 to 1.07)	2.81 (0.66 to 12.02)
≥55	2.22)**	2.83)**		
Ethnicity				
Ethnic majority/ English-speaking	0.91 (0.71 to 1.16)	1.22 (0.92 to 1.62)	1.08 (0.79 to 1.47)	1.39 (0.85 to 2.28)
Ethnic minority/non-English-speaking	Ref	Ref	Ref	Ref
Education				
Low	Ref	Ref	Ref	Ref
Moderate	1.11 (0.89 to 1.40)	1.11 (0.83 to 1.47)	1.00 (0.76 to 1.32)	0.99 (0.71 to 1.37)
High	1.06 (0.81 to 1.39)	1.76 (1.26 to 2.46)**	0.93 (0.68 to 1.25)	0.95 (0.65 to 1.38)
Income				
Low	Ref	Ref	Ref	Ref
Moderate	1.17 (0.91 to 1.50)	0.82 (0.61 to 1.12)	0.85 (0.66 to 1.10)	1.08 (0.73 to 1.61)
High	1.05 (0.82 to 1.34)	0.81 (0.59 to 1.10)	0.94 (0.73 to 1.20)	1.54 (1.09 to 2.18)^*^
No information	1.23 (0.80 to 1.88)	0.68 (0.07 to 6.24)	0.83 (0.53 to 1.28)	0.68 (0.34 to 1.35)
Smoking status				
Weekly	Ref	Ref	Ref	Ref
Daily	1.27 (0.97 to 1.66)	1.04 (0.74 to 1.48)	1.27 (0.98 to 1.65)	1.07 (0.58 to 1.96)
Vaping status				
Daily	0.96 (0.71 to 1.30)	1.16 (0.79 to 1.71)	0.79 (0.62 to 1.01)	0.60 (0.32 to 1.11)
Non-daily	1.28 (0.99 to 1.64)	0.86 (0.58 to 1.26)	0.95 (0.74 to 1.21)	1.25 (0.75 to 2.06)
Non-vaper	Ref	Ref	Ref	Ref
Knowledge of smoking harms^	1.23 (1.07 to 1.40)**	1.24 (1.04 to 1.48)^*^	1.23 (1.09 to 1.40)**	1.88 (1.53 to 2.32)***
Belief re harmfulness of nicotine to health Not at all/slightly Mod/very/extreme/DK	2.27 (1.83 to 2.81)*** Ref	1.53 (1.14 to 2.04)** Ref	2.03 (1.67 to 2.46)*** Ref	1.89 (1.36 to 2.62)*** Ref
Belief re harmfulness of vaping relative to smoking^ Much less harmful Somewhat less Equally/more harmful Don’t know	7.24 (5.18 to 10.14)*** 2.33 (1.88 to 2.88)*** Ref 1.09 (0.77 to 1.52)	7.37 (4.86 to 11.18)*** 1.97 (1.49 to 2.61)*** Ref 0.49 (0.30 to 0.79)**	14.27 (10.69 to 19.04)*** 2.39 (1.92 to 2.97)*** Ref 1.25 (0.88 to 1.78)	13.09 (7.80 to 21.95)*** 3.23 (2.30 to 4.52)*** Ref 1.36 (0.93 to 2.00)

aOR = odds ratio adjusted for the variables in the table; CI = confidence intervals;

*significant at *p* < .05; ** *p* < .01; *** *p* < .001;

^ significant by-country interaction with age group (*p* = .009); knowledge of smoking harms (*p* = .003), and belief re harmfulness of vaping relative to smoking (*p* = .002);

### Sociodemographics

None of the sociodemographic variables were consistently associated with NRT relative harm belief across all countries (see **Table 2**). Association with age group varied by country (*p* < .01). In Canada and the United States, those aged ≥55 were more likely to endorse the belief that NRTs are much less harmful than CCs (adjusted odds ratio [aOR] = 1.65 and 1.93, both *p* < .01; respectively) than those aged 18–24, with Australia showing similar effect but not statistically significant. By contrast, older age groups were no more or less likely to do so in England. For education, those with higher education in the United States were more likely to believe that NRTs are much less harmful, compared to those with lower education (aOR = 1.76, *p* < .01). For income, those with high income in Australia, were more likely to believe that NRTs are much less harmful compared to those with low income (aOR = 1.54, *p* < .05). Gender and ethnicity showed no association with NRT relative harm belief in all countries.

### Smoking and Vaping Status

Across all countries, there was no significant association between NRT relative harm belief and smoking or vaping status.

### Knowledge and Belief About Harms of Smoking, Vaping, and Nicotine

Respondents in all four countries with more knowledge of smoking harms were more likely to hold the belief that NRTs are much less harmful than CCs. However, the effect was significantly stronger in Australia compared to the other countries (AU: aOR = 1.88 vs. CA: aOR = 1.23, United States: aOR = 1.24, and EN: aOR = 1.23; by-country interaction significant at *p* < .01). Across all countries, those who believed that NVPs are much or somewhat less harmful than smoking were more likely to believe that NRTs are much less harmful than CCs. However, there were country differences (p<.01). For example, the effect for the belief that NVPs are much less harmful was significantly stronger in England and Australia than in Canada and the United States (aOR=14.27 and 13.09 vs 7.24 and 7.37, respectively, all *p*’s < .001). Of note in the United States, those who expressed “Don’t Know” when asked about relative harm of NVPs were less likely to believe that NRTs are much less harmful than CCs (aOR = 0.49, *p* < .01). Belief about the harm of nicotine to one’s health was positively and similarly associated with NRT relative harm belief in all countries, that is, those who believed nicotine is not harmful at all or only slightly harmful were more likely to endorse that NRTs are much less harmful than CCs than those who believed nicotine is moderate to extremely harmful or unaware of its harmfulness (aOR range = 1.53–2.27, all *p*’s < .01, see **Table 2**).

Factors conjointly associated with the belief that NRTs are much less harmful than CCs

The overall DTA model performance was acceptable across the four countries (AUC scores range = 0.63–0.66, see Supplementary Table S2). When cross-validated, the individual country model showed both predictive and discriminant validity, with better performance on within-country than between-country test data. The DTA revealed that NVP relative harm belief, nicotine harm belief, knowledge of smoking harms, and sociodemographic factors such as age, education, and gender were the factors that interacted and were conjointly associated with NRT relative harm belief (see Supplementary Figure S2). Of these variables, NVP relative harm belief and age were the only two factors common across all four countries with the rest being country specific.

NVP relative harm belief was also notably the first factor to be selected in all four countries (and hence, the most important factor) for classifying those who smoke into subgroups with high versus low probability of believing NRTs are much less harmful than CCs. In all countries, those who perceived NVPs as less harmful were distinguished from the rest (equal/more harmful/Don’t Know). Country-specific results are presented below in turn for further distinctions which differed by country.

In Canada, among the low NVP relative harm believers, four subgroups were identified (see **Supplementary Figure S2**-A). Like regression results, those believing NVPs are much less harmful (group 1) were more likely to believe NRT is much less harmful than smoking (69%). The remaining who believed NVPs are somewhat less harmful (group 2) were further distinguished by age (nonlinearly) with those aged either 18–24 or 40–54 years being more likely to hold correct NRT relative harm belief (60%). The rest aged 25 to 39 or 55 and older were distinguished further based on their nicotine belief, with those who believed nicotine is no more harmful than slightly being more likely to hold “correct” NRT relative harm belief (55%) versus the remaining cases being less likely (46% correct). Turning to the other arm of high NVP relative harm belief cases, two subgroups were identified based on the conjoint belief about NVP relative harm and nicotine’s harm to health. The first (group 6) was defined by those believing NVPs are equally or more harmful than CCs, or did not know the answer together with the belief that nicotine is at least moderately harmful to health yielding a low probability of correct NRT relative harm belief (37%). The remaining cases (group 5) shared similar NVP relative harm beliefs but thought nicotine was no more than slightly harmful with a near-chance level of correct NRT relative harm belief (53%).

In the United States, among the low NVP relative harm belief cases, four subgroups were identified (**Supplementary Figure S2**-B), defined conjointly with age and gender. Group 1 with 77% correct NRT relative harm belief, consisted of those who perceived NVP as much/somewhat less harmful than smoking and aged 18–40, whereas group 2 were those aged 40 or older who perceived NVPs as much less harmful (66% correct). The remaining cases were further distinguished by gender with male cases (group 3) having near-chance (52%), versus their female counterparts having low (42%), probability of “correct” NRT relative harm perception. Among the other arm of high NVP relative harm believers, three subgroups were identified conjointly with age and education. However, age was nonlinearly associated with NVP relative harm belief to define group 5, characterized by those who perceived NVPs as not less harmful than CCs or were unaware of its harmfulness and aged 25–39 (20% correct). The remaining cases (aged 18–24 or ≤40) were further distinguished by level of education into those with low to moderate education (group 6) having low probability of “correct” NRT relative harm perception (37%) versus the rest (group 7) with near-chance level (52%).

In England, four subgroups were identified (**Supplementary Figure S2**-C) among the right arm of low NVP relative harm belief cases. Like Canada, those who believed NVPs are much less harmful (group 1) were more likely to hold “correct” NRT relative harm perception (66%) with the remaining further distinguished by age, with those aged 18–39 (group 2) also more likely to hold “correct” NRT relative harm perception (58%). The remaining cases (≥40+) were further classified conjointly based on their nicotine harm belief into those who believed nicotine is no more than slightly harmful to one’s health (group 3) with near-chance probability (53% correct) versus the rest (group 4) with low probability (42% correct). For the left arm of high NVP relative harm belief cases, only one group (group 5) was identified by this belief with no other conjoint factor (33% correct).

In Australia, among those with low NVP relative harm belief, three subgroups were identified (**Supplementary Figure S2**-D) with somewhat different pattern of variables defining the identified subgroups from that of the other countries. Group 1 (68% correct) was conjointly defined by those who believed NVPs are less harmful than CCs together with believing nicotine is no more than slightly harmful. The remaining cases were further distinguished by knowledge of smoking harm into those with a smoking harm knowledge score of 1.5 and higher (group 2) with 59% correct NRT relative harm belief versus 47% among those with knowledge score below 1.5. Among the high NVP relative harm believers, four subgroups were distinguishable. Group 4 was conjointly defined by those who believed NVPs are either more or equally harmful than smoking or don’t know and scored more than 0.5 on smoking harm knowledge measure (15% correct). Those with higher scores were further distinguished conjointly by age into those aged 25–54 (34% correct) with the rest (ie, those aged ≥55) further classified by NVP relative harm belief into those believing NVPs are either more or equally harmful than smoking (group 6; 38% correct) versus those who did not know the answer regarding NVP relative harmfulness (group 7; 54% correct).

## Discussion

### Key Findings

This study examined beliefs about the relative harm of NRT to CCs among people who smoke regularly in Canada, the United States, England, and Australia. We found that many respondents from these countries (nearly three-quarters in England, Canada and Australia, and around four-fifths in the United States) were unaware that using NRT is much less harmful compared to cigarettes. This misperception about the relative harmfulness of NRT likely contributes to hesitancy to use an efficacious nicotine medication currently approved for smoking cessation by the governments in all four countries.^13^

Our results indicated that there were both country-common and country-specific factors associated with the belief that NRTs are much less harmful than CCs among people who smoke in the four countries. The most important determinant common to all four countries was the parallel belief in the relative harmfulness of NVPs. This shows a degree of consistency and suggests that NRT and NVPs are evaluated similarly. Beyond this, there were clear country-specific differences as well. Our stress testing suggests that some of these are real, but it would be useful to revalidate them on a subsequent wave of data to see if they are stable. The only other variable having an influence common for all countries, was age. However, its impact varied across countries, and in two of the countries (ie, Canada and the United States), the relationships were not linear, with intermediate groups having different responses to those both younger and older and having influences on different subgroups. Taken together, these findings suggest that their influence may be affected by contextual factors such as differences in tobacco control history/activity contributing to people’s awareness and understanding of harm related to nicotine, smoking, and vaping.

The finding from both analytic methods that those misperceiving NVP relative harms were more likely to also misperceive NRT relative harms in these countries is consistent with that of past research^8,32^ although it may not be causal. Given both products contain nicotine, the association may simply reflect the extent to which people believe that nicotine is the cause of harm. This explanation, however, is likely insufficient given that our regression analysis controlled for people’s beliefs about nicotine's harmfulness. It is worth noting that the regression effect found in England and Australia for the subgroup who believed that NVPs are much less harmful than CCs was much stronger than that in Canada and the United States. This might reflect a greater awareness and understanding of nicotine and its limited contribution to the harmfulness of various nicotine-containing products among people who smoke from these countries, rather than different policy environments for NVPs since England and Australia have very different policies on NVPs.^33^

While the regression analysis also indicated that respondents’ knowledge of smoking harms was another consistent predictor of NRT relative harm perceptions across all four countries, it is notable that the association in Australia was the strongest of the countries suggesting it is an important factor influencing people’s perceptions of NRT in Australia. Its importance in Australia is also highlighted in DTA which showed that it interacted conjointly with other factors to influence people’s NRT relative harm perceptions contributing to the heterogeneity of subgroups with varying levels of understanding of NRT relative harm perceptions.

The finding of country differences from the regression analysis whereby only in the United States and Australia were the subgroup with high education and high income being more likely to be accurate in their NRT relative harm perceptions than their respective counterparts with low education/income is difficult to explain. Nevertheless, this regression finding underscores the need to address this disparity to ensure that people from lower socioeconomic backgrounds in the United States and Australia are able to make informed decisions about the use of NRT for smoking cessation.

The DTA findings highlight the key advantage it has over standard regression in uncovering more complex interactions between predictor variables. While both methods show that belief about the harmfulness of vaping relative to smoking was a strong predictor of belief about the relative harmfulness of NRT, DTA suggested that the effect of this predictor also interacts with other factors, such as age, to conjointly influence people’s perceptions of NRT harms relative to CCs resulting in heterogeneous subgroups with different perceptions. While age was a country-common factor in the DTA, its contribution to the conjoint effect was not necessarily linear as in the regression analysis. In England, this conjoint influence appears to be simpler than that in other countries. Unlike England, for the other countries, the belief about NRT relative harmfulness among the subgroup who misperceived the relative harm of vaping to smoking also appears to be influenced jointly by the presence of other factors, yielding several different subgroups with varying probability of a “correct” perception of NRT relative harmfulness. Of particular value is the identified subgroup in Australia (group 4) characterized by a lack of an accurate understanding of the relative harmfulness of vaping and smoking as these participants were more likely to be misinformed about NRT relative harms and would benefit from corrective interventions. The heterogeneity in findings across countries suggests that in a country with less consistent messaging around nicotine and nicotine-containing products such as Australia^34^ and the United States (becoming more restrictive in NVP policy recently),^35,36^ the influence on relative harm perceptions of NRTs is much more complex and fragmented.

### Finding Implications

Modifiable factors such as knowledge of smoking harms and their understanding of the harmfulness of nicotine and nicotine-containing products can be targeted for intervention to improve understanding of the relative harmfulness of NRT. The identified subgroup information can be used to prioritize and inform the development of effective interventions to address specific needs of various subgroups identified within and between countries to ensure that all people who smoke can make informed choices about the use of this approved quitting method for smoking cessation or harm reduction purposes.^13,37^ For example, to maximize the use of limited public health resources, mass-media education campaigns can be designed to target groups with certain sociodemographic profiles identified as the least informed to improve their understanding of the relative harmfulness of NRT to reduce any barriers to its use. Similarly, healthcare professionals in clinical settings can prioritize people who smoke with this risk profile to receive more thorough assessment and help to address specific knowledge deficit and barriers to NRT use. Given the strong protective effect of knowledge of smoking harms on NRT relative harm perception in all four countries, improving understanding of the health effects of smoking among people who smoke should be a priority, particularly in Australia. In addition, efforts should also be directed towards improving people’s understanding of the absolute and relative harm of nicotine, smoking, and vaping, particularly as an accurate understanding of nicotine harmfulness remains low in all four countries. This would help to ensure that those interested in using NRT to aid quitting can use it according to the recommended duration and dosage to fully benefit from its use.^5,12,14^ Past research indicated that use of a fact sheet or brief nicotine messaging intervention, if well-designed, could help to correct nicotine risk misperceptions.^38–40^

### Strengths and Limitations

Study strengths included cross-country comparison, large broadly representative sample of people who smoke, and use of complementary analytic strategies to provide additional insights. Several study limitations warrant some caution. First, the study design does not allow for any causal inferences. Second, findings may not generalize beyond the four high-income countries studied although some consistencies in findings across the four countries and analytic methods are reassuring. Third, the measure used to quantify people’s perceptions of smoking health consequences was not comprehensive and intended as an approximate global measure of people’s awareness of the breadth of different types of smoking harms. Moreover, nuanced information was potentially lost by summing across four items and a different way of quantifying harm perceptions of smoking could change the results or decision-tree hierarchies. Fourth, it is unclear how belief about the relative harmfulness of NRT compared to CCs might be influenced by health professionals’ advice and recommendation on the use of NRTs and personal smoking cessation history. Fifth, the relatively small sample size may have contributed to the lower-than-ideal decision-tree model performance. Country-specific DTA results would need confirmation from future research with larger samples.

## Conclusions

Among people who smoke in Canada, the United States, England, and Australia, their understanding of the harm of vaping as compared to smoking appears to be the single most important and common modifiable factor that are both individually and conjointly associated with their understanding of the relative harm of NRT products. Knowledge of smoking harms is another common modifiable factor that has a strong individual association in all four countries, but its conjoint association appears to vary across countries. The individual and conjoint associations of other factors such as sociodemographic background and understanding of whether nicotine is harmful to one’s health also show country variations. The observation that many people who smoke are misinformed about the harmfulness of nicotine and NRT underscores the need for further corrective interventions to encourage adoption of lower-risk nicotine alternatives to cigarettes.

## Supplementary Material

A Contributorship Form detailing each author’s specific involvement with this content, as well as any supplementary data, are available online at https://academic.oup.com/ntr.

ntad075_suppl_Supplementary_Materials

## Data Availability

In each country participating in the international Tobacco Control Policy Evaluation (ITC) Project, the data are jointly owned by the lead researcher(s) in that country and the ITC Project at the University of Waterloo. Data from the ITC Project are available to approved researchers 2 years after the date of issuance of cleaned data sets by the ITC Data Management Center. Researchers interested in using ITC data are required to apply for approval by submitting an International Tobacco Control Data Repository (ITCDR) request application and subsequently to sign an ITCDR Data Usage Agreement. The criteria for data usage approval and the contents of the Data Usage Agreement are described online (http://www.itcproject.org).
