# Supplementary material for: Individual and Conjoint Factors Associated With Beliefs About the Harmfulness of Nicotine Replacement Therapies Relative to Combustible Cigarettes Among People Who Smoke: Findings From the 2020 ITC Four Country Smoking and Vaping Survey
Source: Nicotine Tob Res. 2023 May 17;25(9):1594–602. doi: 10.1093/ntr/ntad075 (PMC10439491; doi:10.1093/ntr/ntad075)
Supplement: ntad075_suppl_Supplementary_Materials [file ntad075_suppl_supplementary_materials.docx]

Training data

70% of total sample

CA=1843; US=1219;

EN=2140; AU=848

Testing data

30% within-country (CA=790; US=520;

EN=917; AU=365)

& 100% cross-country total sample

Selecting the best model from K models and verified using Testing data

Final training data

after class balancing

CA=932; US=568; EN=1088; AU=531

90% for Training

10% for Validation

K-Fold Validation

(Repeating this process for K=10 times)

Training data

after removing missing data

CA=1787; US=1192; EN=2057; AU=832

Survey Sample

Canada [CA] n=2633; United States [US] n=1739; England [EN] n=3057; Australia [AU] n=1213

Model for predicting risk perception

Supplementary Figure S1. Flowchart showing the process involved and the samples used in decision tree analysis.


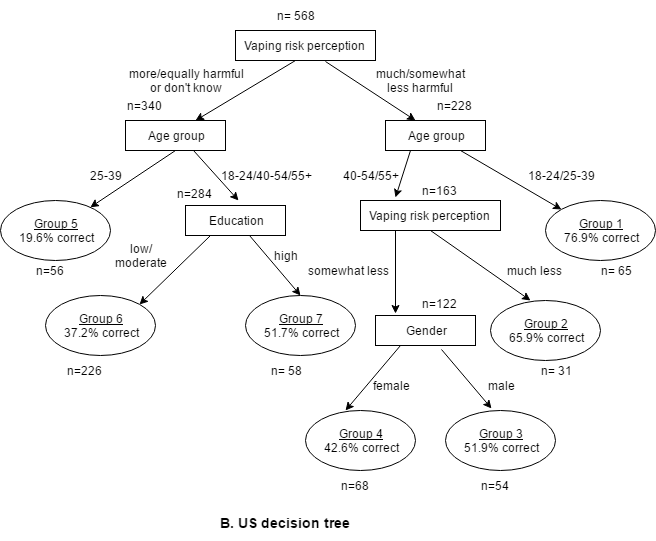

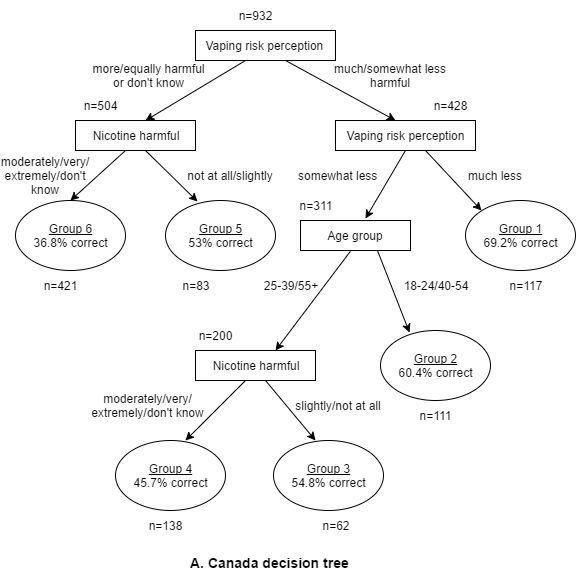


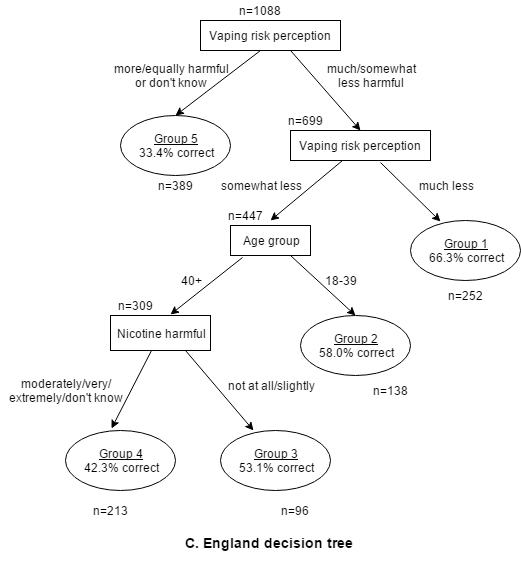

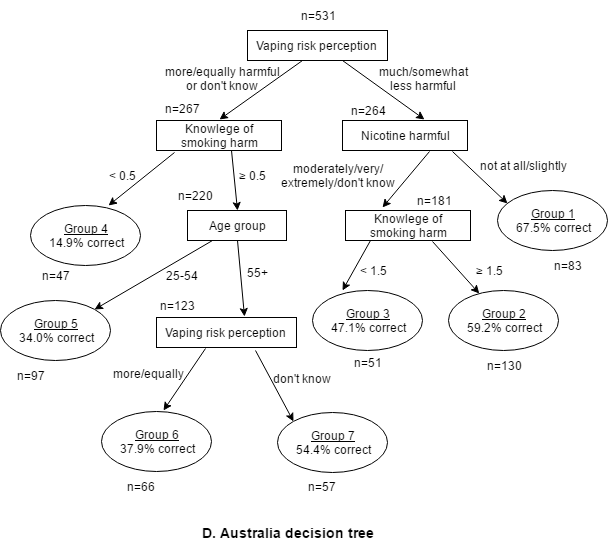


Supplementary Figure S2 (A-D). Decision-tree diagram showing factors conjointly associated with harm perception of nicotine replacement therapy products relative to combustible cigarettes in each country.

*Factor relative importance.* The relative importance of the factors for outcome prediction is indicated by their ranked order on the tree nodes with those higher up being more important than those lower down the tree branches. For example, in England, vaping risk perception was the most important factor followed by age, with nicotine harm belief being the least important factor.

*Factor response splitting.* At each tree node, the decision-tree algorithm will split the factor responses optimally to achieve maximum prediction accuracy; responses of categorical factor can be split and combined in a nonlinear manner.

Correct=believed nicotine replacement products are much less harmful than combustible cigarette smoking.

Supplementary Table S1. Logistic regression results showing unadjusted and adjusted association of different factors with belief about nicotine replacement therapy products being much less harmful than combustible cigarettes among people who currently smoke daily/weekly.

| **Variables** | **Canada**  **N=2549** | | **US**  **N=1706** | | **England**  **N=2935** | | **Australia**  **N=1183** | |
| --- | --- | --- | --- | --- | --- | --- | --- | --- |
|  | uOR (95% CI) | aOR (95% CI) | uOR (95% CI) | aOR (95% CI) | uOR (95% CI) | aOR (95% CI) | uOR (95% CI) | aOR (95% CI) |
| Gender  Female  Male | 0.92 (0.77-1.09)  Ref | 1.06 (0.88-1.29)  Ref | 0.80 (0.64-0.99)  Ref | 0.88 (0.69-1.13)  Ref | 0.87 (0.74-1.02)  Ref | 0.96 (0.80-1.15)  Ref | 1.02 (0.80-1.29)  Ref | 1.17 (0.88-1.54)  Ref |
| Age group^  18-24  25-39  40-54  55+ | Ref  1.02 (0.79-1.33)  1.16 (0.91-1.49)  1.19 (0.94-1.52) | Ref  1.03 (0.77-1.38)  1.39 (1.04-1.86)*  1.65 (1.23-2.22)** | Ref  1.30 (0.89-1.90)  1.22 (0.85-1.74)  1.45 (1.08-1.97)* | Ref  1.32 (0.87-2.02)  1.47 (0.97-2.23)  1.93 (1.32-2.83)** | Ref  0.81 (0.64-1.03)  0.98 (0.78-1.22)  0.94 (0.76-1.16) | Ref  0.87 (0.66-1.15)  0.92 (0.71-1.20)  0.82 (0.63-1.07) | Ref  1.57 (0.42-5.86)  1.85 (0.51-6.77)  1.85 (0.51-6.70) | Ref  1.72 (0.39-7.55)  2.44 (0.57-10.48)  2.81 (0.66-12.02) |
| Ethnicity  Ethnic majority or English-speaking  Ethnic minority or non-English-speaking | 1.01 (0.81-1.26)  Ref | 0.91 (0.71-1.16)  Ref | 1.29 (1.00-1.66)*  Ref | 1.22 (0.92-1.62)  Ref | 1.11 (0.86-1.43)  Ref | 1.08 (0.79-1.47)  Ref | 1.75 (1.14-2.68)*  Ref | 1.39 (0.85-2.28)  Ref |
| Education  Low  Moderate  High | Ref  1.21 (0.98-1.49)  1.14 (0.90-1.43) | Ref  1.11 (0.89-1.40)  1.06 (0.81-1.39) | Ref  1.14 (0.87-1.48)  1.87 (1.40-2.50)*** | Ref  1.11 (0.83-1.47)  1.76 (1.26-2.46)** | Ref  1.01 (0.79-1.28)  0.86 (0.66-1.12) | Ref  1.00 (0.76-1.32)  0.93 (0.68-1.25) | Ref  1.21 (0.91-1.63)  1.24 (0.90-1.69) | Ref  0.99 (0.71-1.37)  0.95 (0.65-1.38) |
| Income  Low  Moderate  High  No information | Ref  1.14 (0.91-1.43)  1.05 (0.84-1.30)  0.99 (0.67-1.46) | Ref  1.17 (0.91-1.50)  1.05 (0.82-1.34)  1.23 (0.80-1.88) | Ref  0.97 (0.74-1.29)  1.04 (0.80-1.35)  0.56 (0.07-4.67) | Ref  0.82 (0.61-1.12)  0.81 (0.59-1.10)  0.68 (0.07-6.24) | Ref  0.95 (0.76-1.19)  0.99 (0.80-1.22)  0.74 (0.51-1.07) | Ref  0.85 (0.66-1.10)  0.94 (0.73-1.20)  0.83 (0.53-1.28) | Ref  0.98 (0.69-1.41)  1.53 (1.14-2.05)**  0.67 (0.38-1.21) | Ref  1.08 (0.73-1.61)  1.54 (1.09-2.18)*  0.68 (0.34-1.35) |
| Smoking status  Weekly  Daily | Ref  1.08 (0.86-1.35) | Ref  1.27 (0.97-1.66) | Ref  0.97 (0.72-1.32) | Ref  1.04 (0.74-1.48) | Ref  1.22 (0.98-1.52) | Ref  1.27 (0.98-1.65) | Ref  0.77 (0.47-1.26) | Ref  1.07 (0.58-1.96) |
| Vaping status  Daily  Non-daily  Non-vaper | 1.37 (1.07-1.75)*  1.36 (1.10-1.68)**  Ref | 0.96 (0.71-1.30)  1.28 (0.99-1.64)  Ref | 1.43 (1.05-1.95)*  1.03 (0.74-1.42)  Ref | 1.16 (0.79-1.71)  0.86 (0.58-1.26)  Ref | 1.32 (1.08-1.61)**  1.14 (0.93-1.40)  Ref | 0.79 (0.62-1.01)  0.95 (0.74-1.21)  Ref | 1.98 (1.22-3.20)**  1.55 (1.02-2.37)*  Ref | 0.60 (0.32-1.11)  1.25 (0.75-2.06)  Ref |
| Knowledge of smoking harms^ | 1.14 (1.01-1.29)* | 1.23 (1.07-1.40)** | 1.20 (1.02-1.41)* | 1.24 (1.04-1.48)* | 1.37 (1.23-1.53)*** | 1.23 (1.09-1.40)** | 1.78 (1.49-2.13)*** | 1.88 (1.53-2.32)*** |
| Belief re harmfulness of nicotine to health  Not at all/slightly  Mod/very/extreme/DK | 2.57 (2.11-3.12)***  Ref | 2.27 (1.83-2.81)***  Ref | 1.83 (1.42-2.37)***  Ref | 1.53 (1.14-2.04)**  Ref | 2.54 (2.14-3.01)***  Ref | 2.03 (1.67-2.46)***  Ref | 1.94 (1.48-2.56)***  Ref | 1.89 (1.36-2.62)***  Ref |
| Belief re harmfulness of vaping relative to smoking^  Much less harmful  Somewhat less  Equally/more harmful  Don’t know | 7.41 (5.44-10.09)***  2.32 (1.90-2.84)***  Ref  1.12 (0.81-1.54) | 7.24 (5.18-10.14)***  2.33 (1.88-2.88)***  Ref  1.09 (0.77-1.52) | 7.28 (4.95-10.70)***  2.03 (1.55-2.66)***  Ref  0.52 (0.33-0.83)** | 7.37 (4.86-11.18)***  1.97 (1.49-2.61)***  Ref  0.49 (0.30-0.79)** | 15.78 (12.02-20.73)***  2.65 (2.15-3.28)***  Ref  1.18 (0.84-1.65) | 14.27 (10.69-19.04)***  2.39 (1.92-2.97)***  Ref  1.25 (0.88-1.78) | 10.39 (6.64-16.24)***  3.33 (2.41-4.60)***  Ref  1.15 (0.80-1.66) | 13.09 (7.80-21.95)***  3.23 (2.30-4.52)***  Ref  1.36 (0.93-2.00) |

Note: uOR, unadjusted odds ratio; aOR, odds ratio adjusted for all the other variables in the table; CI, confidence intervals;

* significant at p<.05; ** p<.01; *** p<.001;

^ significant by-country interaction with age group (p=0.009); knowledge of smoking harms (p=0.003), and belief re harmfulness of vaping relative to smoking (p=0.002);

Supplementary Table S2. Decision-tree model performance when cross-validated on independent within and between country test data.

| Country model | Cross-validation data | N | TP | FP | TN | FN | Sensitivity | Specificity | AUC |
| --- | --- | --- | --- | --- | --- | --- | --- | --- | --- |
| Canada (CA) | **CA (30% test data)** | **790** | **142** | **208** | **366** | **74** | **0.65** | **0.63** | **0.64** |
|  | US (100% data) | 1730 | 157 | 323 | 1008 | 242 | 0.39 | 0.75 | 0.57 |
|  | EN (100% data) | 3052 | 515 | 645 | 1561 | 331 | 0.60 | 0.70 | 0.65 |
|  | AU (100% data) | 1213 | 163 | 167 | 647 | 236 | 0.40 | 0.79 | 0.60 |
| United States (US) | **US (30% test data)** | **520** | **67** | **138** | **271** | **44** | **0.60** | **0.66** | **0.63** |
|  | CA | 2630 | 203 | 471 | 1387 | 447 | 0.31 | 0.74 | 0.56 |
|  | EN | 3052 | 279 | 620 | 1525 | 441 | 0.38 | 0.71 | 0.60 |
|  | AU | 1213 | 78 | 93 | 711 | 306 | 0.20 | 0.88 | 0.55 |
| England (EN) | **EN (30% test data)** | **917** | **168** | **220** | **442** | **87** | **0.65** | **0.66** | **0.66** |
|  | CA | 2630 | 323 | 402 | 1509 | 396 | 0.44 | 0.78 | 0.61 |
|  | US | 1730 | 149 | 215 | 1116 | 250 | 0.37 | 0.83 | 0.60 |
|  | AU | 1213 | 159 | 114 | 700 | 240 | 0.39 | 0.85 | 0.62 |
| Australia (AU) | **AU (30% test data)** | **365** | **71** | **75** | **176** | **43** | **0.62** | **0.70** | **0.66** |
|  | CA | 2630 | 299 | 384 | 1527 | 420 | 0.41 | 0.79 | 0.60 |
|  | US | 1730 | 123 | 218 | 1113 | 276 | 0.30 | 0.83 | 0.57 |
|  | EN | 3052 | 467 | 650 | 1556 | 379 | 0.55 | 0.70 | 0.62 |

Note: TP, true positive; FP, false positive; TN, true negative; FN, false negative; AUC, Area Under the Curve; AUC value can range from 0.50 to 1.00 where 0.50 indicating model prediction is no better than chance and 1.00 indicating perfect prediction; CA, Canada; US, United States; EN, England; AU, Australia;
